# Genotypes, Recombinant Forms, and Variants of Norovirus GII.4 in Gipuzkoa (Basque Country, Spain), 2009–2012

**DOI:** 10.1371/journal.pone.0098875

**Published:** 2014-06-03

**Authors:** Ainara Arana, Gustavo Cilla, Milagrosa Montes, María Gomariz, Emilio Pérez-Trallero

**Affiliations:** 1 Servicio de Microbiología, Hospital Universitario Donostia-Instituto de Investigación Biodonostia, San Sebastián, Spain; 2 Centro de Investigación Biomédica en Red de Enfermedades Respiratorias (CIBERES), San Sebastián, Spain; 3 Departamento de Medicina Preventiva y Salud Pública, Facultad de Medicina, Universidad del País Vasco (UPV/EHU), San Sebastián, Spain; Centers for Disease Control and Prevention, United States of America

## Abstract

**Background:**

Noroviruses (NoVs) are genetically diverse, with genogroup II—and within it—genotype 4 (GII.4) being the most prevalent cause of acute gastroenteritis worldwide. The aim of this study was to characterize genogroup II NoV causing acute gastroenteritis in the Basque Country (northern Spain) from 2009–2012.

**Methods:**

The presence of NoV RNA was investigated by reverse transcriptase-polymerase chain reaction (RT-PCR) in stool specimens from children younger than 15 years old with community-acquired acute gastroenteritis, and from hospitalized adults or elderly residents of nursing homes with acute gastroenteritis. For genotyping, the open reading frames ORF1 (encoding the polymerase) and ORF2 (encoding the major capsid protein) were partially amplified and sequenced. Recombinant strains were confirmed by PCR of the ORF1/ORF2 junction region.

**Results:**

NoV was detected in 16.0% (453/2826) of acute gastroenteritis episodes in children younger than 2 years, 9.9% (139/1407) in children from 2 to 14 years, and 35.8% (122/341) in adults. Of 317 NoVs characterized, 313 were genogroup II and four were genogroup I. The GII.4 variants Den Haag-2006b and New Orleans-2009 predominated in 2009 and 2010–2011, respectively. In 2012, the New Orleans-2009 variant was partially replaced by the Sydney-2012 variant (GII.Pe/GII.4) and New Orleans-2009/Sydney-2012 recombinant strains. The predominant capsid genotype in all age groups was GII.4, which was the only genotype detected in outbreaks. The second most frequent genotype was GII.3 (including the recently described recombination GII.P16/GII.3), which was detected almost exclusively in children.

**Conclusion:**

Nine different genotypes of NoV genogroup II were detected; among these, intergenotype recombinant strains represented an important part, highlighting the role of recombination in the evolution of NoVs. Detection of new NoV strains, not only GII.4 strains, shortly after their first detection in other parts of the world shows that many NoV strains can spread rapidly.

## Introduction

Norovirus (NoV) is a frequent cause of sporadic acute gastroenteritis (AGE) and is the main cause of gastroenteritis epidemics worldwide in all age groups [1], [2]. In children younger than 5 years, NoV is the second cause of severe AGE after rotavirus [1], being the main cause in countries which have implemented rotavirus vaccination programs [3]. Outbreaks of AGE due to NoV are common in semi-enclosed environments such as hospitals, nursing homes, cruise ships and army barracks and can affect large numbers of people [2]. These viruses are transmitted from person to person through the fecal-oral route, aerosolized vomit or contact with contaminated surfaces, as well as through contaminated food and water, and are the main cause of outbreaks of diarrhea caused by food [2], [4].

NoVs are a genus within the *Caliciviridae* family and are non-enveloped viruses whose genome has a single-stranded, positive sense, RNA that includes three open reading frames (ORF). ORF1 codifies for six non-structural proteins, including RNA-dependent RNA polymerase (RdRp). ORF2 codifies the major capsid protein (VP1) and ORF3 the minor capsid protein (VP2) [5]. The ORF1 and ORF2 sequences form the basis of the currently proposed NoV genotyping system [6]. Based on the genetic sequence of VP1, five NoV genogroups (GI-GV) have been described, of which only GI and GII, and more rarely GIV, infect humans [5]. Nine genotypes have been proposed within genogroup I and 22 within genogroup II [7], with GII.4 being the most prevalent genotype worldwide [8], [9]. The virus is highly versatile and new strains frequently arise due to antigenic drift in the VP1 and to genetic recombination between preexisting NoV strains [10], [11]. The aim of the study was to describe the circulation of genogroup II NoV in a region of northern Spain (Gipuzkoa, Basque Country) between 2009 and 2012.

## Methods

### Ethics Statement

In the present study no human experimentation was conducted, with all studies carried out on norovirus strains. The results were obtained from samples that had previously been sent to our laboratory for routine virological diagnosis of gastroenteritis. The data referred to are associated with norovirus strains, with no patient information used other than age and association or not with outbreaks or nosocomial infection. Publication of these results was approved by the ‘Ethical Committee for Clinical Research of the Health Area of Gipuzkoa’.

### Study population

Between January 2009 and December 2012, the presence of NoV was investigated in fecal samples from children younger than 15 years with sporadic AGE attended in primary care clinics and at the emergency department of Hospital Universitario Donostia. The presence of NoV was also investigated in adults affected by outbreaks of AGE (three or more subjects coinciding in time and place) occurring in nursing homes or hospitals, and in other adults suffering AGE during their hospital admission but who did not apparently form part of an outbreak.

### Norovirus detection

Stool samples were resuspended in B199 medium (Sigma-Aldrich, USA), with daily extraction of viral RNA (NucliSENS Easy-Mag system, bio-Mèrieux SA, Marcy l'Etoile, France). Then, cDNA was transcribed using random hexamers and the M-MuLV enzyme (USB Corp., Cleveland, OH, USA). Viral detection was carried out through polymerase chain reaction (PCR), using the Ni and E3 primers, which amplify a region of 113 bp of the polymerase gene [12]. An aliquot of stool samples resuspended in B199 was stored at −80°C for subsequent analysis.

### Genotyping

Genotyping was performed in 56%, 67%, 57% and 86% of the NoV detected in 2009, 2010, 2011 and 2012 respectively. Only the first NoV detected was included if several positive samples were available from a single patient in a three-month period. Samples for genotyping were analyzed using two PCRs directed at region A of the polymerase gene (ORF1, 319 bp) and at region C of the capsid gene (ORF2, 317 bp), using the primers p289IUB/p290IUB [13] and JV24/JV21 [13], [14], respectively. PCR was carried out in a final 25 µl-volume using the 9800 Fast Thermal Cycler (Applied Biosystem, USA), with annealing temperatures of 42°C for polymerase and of 49°C for capsid. Amplified products were sequenced using the ABI3730xl DNA Analyzer (Applied Biosystems, USA). The genogroup, genotype and variant were assigned by using the NoV Noronet typing tool (http://www.rivm.nl/mpf/norovirus/typingtool) [6] and the strains were named by indicating the genotype of the polymerase followed by that of the capsid [7].

### Detection and analysis of recombinant strains

To analyze recombinant strains (capsid and polymerase of a distinct genotype), a region of 1095 bp in the ORF1/ORF2 junction of the viral genome was amplified using the p289IUB [13] and G2SKR primers [15]. PCR was performed in a final 50 µl-volume with an annealing temperature of 51°C, and the amplicons were sequenced and analyzed using the above-mentioned tools. Sequences representative of the main variants of GII.4 and of the recombinant strains detected in this study were deposited in GenBank (accession numbers KJ156623, KJ504381-KJ504428). A phylogenetic analysis of the partial capsid gene of GII.3 strains detected throughout the study and of the partial polymerase gene associated GII.P16 was conducted in Mega 6 through the Maximun Likelihood method using the best model, Kimura 2-parameter+G, as determined also in Mega 6 using the Bayesian information criterion, with 1000 bootstrap replications for branch support. Discrete variables were analyzed by the chi square test using the GraphPad Instat ver 3.05 software (GraphPad Software Inc.La Jolla, CA, USA).

## Results

NoV was detected in 14.0% (592/4233) of children younger than 15 years with sporadic AGE, of which 56.9% (n = 337) were boys. A total of 453 NoV-positive episodes occurred in infants and children younger than 2 years (16.0%, n = 2826) and 139 occurred in children from 2 to 14 years (9.9%, n = 1407). There was no difference in the percentage of positive samples between patients attended at the emergency room of the hospital (13.2%, 212/1610) and children attended in primary care (14.5%, 380/2623)(p = 0.2). Of 341 adults with AGE (39.3% males), 167 were patients affected in outbreaks of AGE in nursing homes or hospitals being NoV detected in 81 (46.6%). In addition, 174 were hospitalized adults with AGE not apparently included in any hospital outbreak, among which NoV was detected in 41 (23.6%).

In total, 714 samples were NoV-positive. Genotyping was performed in 466 strains (65.3%) and the genotype of the capsid and/or polymerase was obtained in 317, of which 313 (98.7%) belonged to genogroup II and four to genogroup I.

### Genetic analysis of norovirus genogroup II

#### Genotypes

The genotype was obtained from two genes (polymerase and capsid) in 257 specimens (82.1%), from the polymerase gene alone in 18 (5.8%) and from the capsid alone in 38 (12.1%). The most prevalent NoVs were GII.4 (65.8% of the strains genotyped with both genes), GII.Pe/GII.4 (14.4%), GII.P21/GII.3 (8.9%) and GII.P16/GII.3 (5.8%) (Table 1).

**Table 1:** **Total number of norovirus genogroup II detected in Gipuzkoa (Basque Country, Spain), 2009–2012.**

	2009	2010	2011	2012	Total
Genotype	n (%)	n (%)	n (%)	n (%)	n (%)
**ORF1/ORF2**					
GII.P4/GII.4	80 (73.4)	37 (67.3)	41 (83.7)	11 (11.0)	169 (54.0)
*Den Haag-2006b*	*76 (95.0)*	*2 (5.4)*	*0*	*0*	*78 (46.1)*
*Apeldoorn-2007*	*1 (1.2)*	*1 (2.7)*	*0*	*0*	*2 (1.2)*
*New Orleans-2009*	*2 (2.5)*	*30 (81.1)*	*37 (90.2)*	*6 (54.5)*	*75 (44.4)*
*New Orleans/Sydney* a	*0*	*0*	*0*	*5 (45.5)*	*5 (3.0)*
*not assigned*	*1(1.2)*	*4 (10.8)*	*4 (9.8)*	*0*	*9 (5.3)*
GII.Pe/GII.4	4 (3.7)	0	0	33 (33.0)	37 (11.8)
*Osaka-2007*	*3 (75.0)*	*0*	*0*	*0*	*3 (8.1)*
*Sydney-2012*	*0*	*0*	*0*	*33 (100.0)*	*33 (89.2)*
*not assigned*	*1 (25.0)*	*0*	*0*	*0*	*1 (2.7)*
GII.P21/GII.3	11 (10.1)	0	0	12 (12.0)	23 (7.3)
GII.P16/GII.3	0	1 (1.8)	0	14 (14.0)	15 (4.8)
GII.P2/GII.2	1 (0.9)	0	2 (4.1)	4 (4.0)	7 (2.2)
GII.P7/GII.6	0	0	0	2 (2.0)	2 (0.6)
GII.Pg/GII.12	1 (0.9)	0	0	1 (1.0)	2 (0.6)
GII.P4/GII.12	0	1 (1.8)	0	0	1 (0.3)
GII.P16/GII.13	0	1 (1.8)	0	0	1 (0.3)
**ORF1** b					
GII.P4	2 (1.8)	4 (7.3)	0	0	6 (1.9)
GII.P21	0	1 (1.8)	0	1 (1.0)	2 (0.6)
GII.Pe	0	0	0	2 (2.0)	2 (0.6)
GII.P2	2 (1.8)	0	0	3 (3.0)	5 (1.6)
GII.P7	0	0	0	2 (2.0)	2 (0.6)
GII.P16	0	0	0	1 (1.0)	1 (0.3)
**ORF2** c					
GII.4	5 (4.6)	8 (14.6)	4 (8.2)	5 (5.0)	22 (7.0)
*Den Haag-2006b*	4 (80.0)	1 (12.5)			5 (22.7)
*New Orleans-2009*		2 (25.0)	4 (100.0)	1 (20.0)	7 (31.8)
*Sydney-2012*				3 (60.0)	3 (13.6)
*not assigned*	1 (20.0)	5 (62.5)		1 (20.0)	7 (31.8)
GII.3	3 (2.8)	0	0	8 (8.0)	11 (3.5)
GII.6	0	1 (1.8)	1 (2.0)	1 (1.0)	3 (1.0)
GII.13	0	1 (1.8)	1 (2.0)	0	2 (0.6)
**Total**	**109**	**55**	**49**	**100**	**313**

Annual distribution of genotypes according to the polymerase RpRd (ORF1) and capsid (ORF2) genes.

a Recombinant strain (New Orleans-2009/Sydney-2012).

b ORF2 not determined (negative PCR of the capsid genotype).

c ORF1 not determined (negative PCR of the polymerase genotype).

#### GII.4 Variants

Of 228 GII.4 strains in which the capsid gene was analyzed, 83 corresponded to the Den Haag-2006b strain (36.4%), 82 to New Orleans-2009 (36.0%), 41 to Sydney-2012 (18.0%), three to Osaka-2007 (1.3%) and two to Apeldoorn-2007 (0.9%). There were 17 strains (7.4%) that could not be assigned to a specific variant.

#### Recombinant strains

Of the total number of strains genotyped with two genes, 31.5% (81/257) corresponded to suspected intergenotype recombinant strains (genotypes of a discordant capsid and polymerase). Notable among these were GII.Pe/GII.4 (n = 37, 45.7%), GII.P21/GII.3 (n = 23, 28.4%) and GII.P16/GII.3 (n = 15, 18.5%). In fact, seven of the nine different genotypes detected, were intergenotype recombinant strains. A PCR of the ORF1/ORF2 junction region of 35 suspected intergenotype recombinant strains confirmed the recombination in all these strains (nine GII.Pe/GII.4, 11 GII.P21/GII.3, 13 GII.P16/GII.3, one GII.P16/GII.13 and one GII.P7/GII.6).

**Figure 1:**
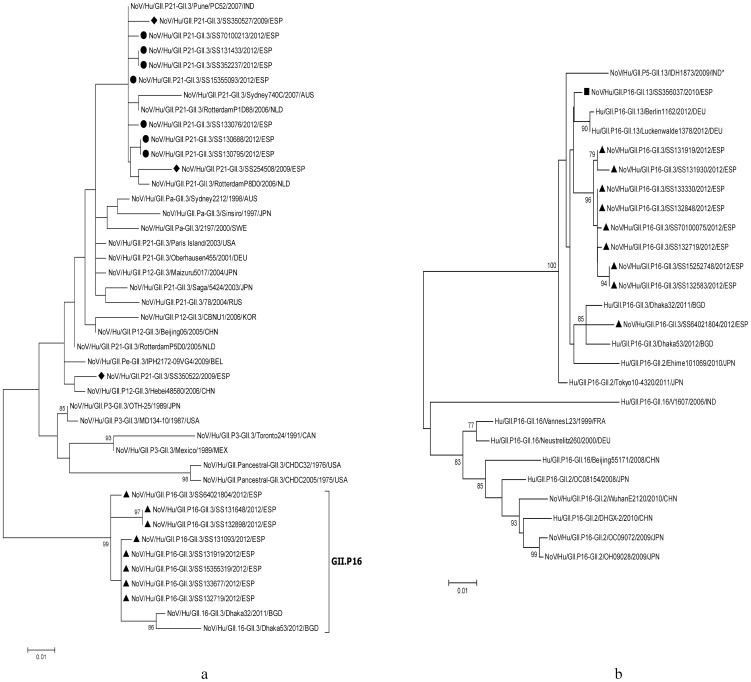
Phylogenetic analyses of GII.3 and GII.P16 norovirus recombinant strains. a) partial capsid (ORF2) GII.3 gene (259 nt) and b) partial polymerase (ORF1) GII.P16 gene (719 nt), from distinct patients with acute gastroenteritis, detected in Gipuzkoa (Basque Country, Spain) in 2009–2012, compared with some representative strains. The trees were constructed in Mega 6 through the Maximun Likelihood method using the best model, Kimura 2-parameter+G, as determined also in Mega 6 using the Bayesian information criterion, with 1000 bootstrap replications for branch support. Bootstrap values >75% are shown. Scale bars indicate the number of substitutions per nucleotide position. Norovirus strains detected in this study are marked with the following symbols: ▴, GII.P16-GII.3; ▪, GII.P16-GII.13; ♦, GII.P21-GII.3 detected in 2009; •, GII.P21-GII.3 detected in 2012. a.- Capsid (ORF2). b.- Polymerase (ORF1). Footnote (figure 1b). *When this sequence was analyzed in the Noronet typing tool, was classified as GII.P16/GII.13.

The partial capsid sequence analysis of the GII.3 NoVs showed that strains associated with GII.P16 polymerase segregated in a cluster, the identity of the intracluster nucleotides being >98%, and nucleotide identity with GII.3 strains associated to GII.P21 circulating the same year being 91.7–93.4% (Figure 1a). On the other hand, analysis of the partial polymerase gene of GII.P16 NoV strains segregated them in two well defined clusters, according to the associated capsid: one grouping the GII.2 and GII.16 strains and the other grouping the recently described recombinations with GII.3 and GII.13 strains, as well as two GII.2 strains detected in Japan in 2010 and 2011 (Figure 1b).

### Dynamics of viral circulation

**Figure 2:**
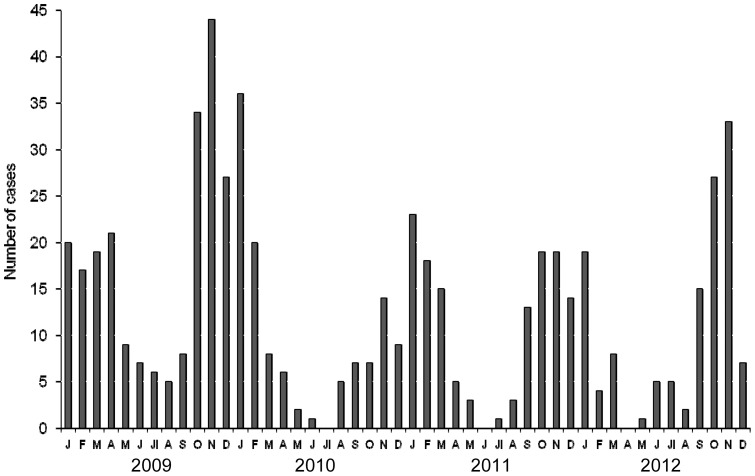
Seasonal circulation of norovirus in children. Monthly distribution of community-acquired acute gastroenteritis episodes due to norovirus in children younger than 15 years diagnosed in Gipuzkoa (Basque Country, Spain), 2009–2012.

**Figure 3:**
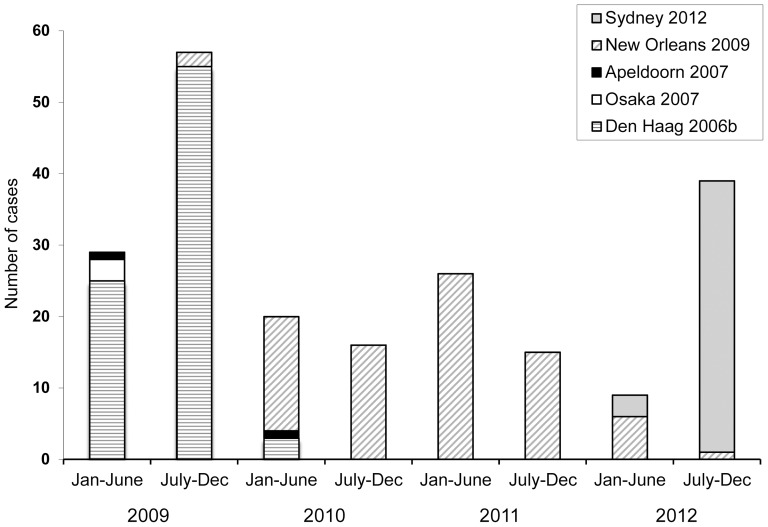
Circulation of genogroup II genotype 4 norovirus variants (GII.4). Six-monthly distribution of GII.4 variants, according to the capsid gene, detected in Gipuzkoa (Basque Country, Spain), 2009–2012.

NoV was detected in all the months of the study except June 2011, and there were no cases among persons younger than 15 years in July 2010 and April 2012 (Figure 2). Overall, the highest number of cases was observed in fall and winter. The temporal distribution of GII.4 variants showed that Den Haag-2006b strains predominated in 2009 and were replaced by New Orleans-2009 strains in 2010; these strains also predominated in 2011 and the first six months of 2012. After February 2012, strains of the new Sydney-2012 variant (GII.Pe/GII.4) were detected and were the main circulating strains in the second half of that year (Figure 3). After July 2012, five strains were detected showing recombination between the Sydney-2012 and New Orleans-2009 variants (New Orleans-2009/Sydney-2012, GII.P4/GII.4), representing 13.9% of the GII.4 strains detected in that semester (July-December 2012). Strains showing the capsid gene GII.3, represented the second most frequent genotype after GII.4, and circulated mainly in 2009 and 2012 (Table 1).

By age groups, between 2009 and 2011, GII.4 strains Den Haag-2006b and New Orleans-2009, predominated in all age groups, accounting for 85.1% (86/101) of the strains detected in children younger than 2 years, 78.3% (18/23) of those in children from 2 to 14 years, and 96.4% (54/56) of those in adults, respectively. In 2012, GII.Pe/GII.4 strains (Sydney-2012) were those most frequently detected in all age groups, accounting for 37.9% (22/58) in children younger than 2 years, 54.5% (6/11) in children from 2 to 14 years and 62.5% (5/8) in adults. Most GII.3 capsid strains (n = 49) were detected in persons younger than 14 years, both in the group less than 2 years (41/182, 22.5%) and in that of 2 to 14 years (7/43, 16.3%). Only one GII.3 strain was detected in one adult (GII.P16/GII.3).

#### Adult infections

Thirty-three outbreaks of AGE were investigated in adults from nursing homes or hospitals and 19 (57.6%) were attributed to NoV. Of these outbreaks, 15 occurred in cold months, 11 of them in fall-winter 2009–2010. The remaining four outbreaks occurred in spring. The percentage of outbreaks attributed to NoV was similar in nursing homes (16/27, 59.3%) and in nosocomial outbreaks (3/6, 50.0%), and there were no outbreaks with mixed etiology (NoV and other enteropathogen). The 16 NoV outbreaks analyzed were caused by GII.4 NoV strains. Among those adults suffering AGE during hospital admission but not apparently included in any AGE outbreak, the capsid genotype GII.4 associated with polymerase genotypes GII.P4 (n = 15) and GII.Pe (n = 5) were the only combinations detected.

## Discussion

In this study, NoV was detected in 14% of sporadic AGE episodes in children, in line with data obtained in studies performed in other countries, including Spain, which have reported figures of 5%–36% in patients attending primary care or hospital emergency rooms due to sporadic AGE [14], [16]–[18]. In addition, NoV was a frequent cause of AGE outbreaks in nursing homes and hospitals, causing more than 50% of the outbreaks studied; this finding was not unexpected, given the major role of NoV as a cause of outbreaks in institutions [2]. AGE caused by NoV was detected in all seasons, but was more frequent in cold months (fall-winter), as reported in other countries with a temperate climate [19]. The highest incidence of infection coincided with the expansion of new viral variants, which occurred in the cold months. Of the 19 NoV outbreaks detected in adults, 15 occurred in fall-winter, 11 of them in 2009–2010.

Most genogroup II NoV strains detected in this study belonged to the capsid genotypes GII.4 and GII.3 (77.3% and 16.6%, respectively), which are the most prevalent strains globally in children with sporadic AGE [9], [20]–[22]. The available information on causal genotypes in Spain is scarce, except for those causing outbreaks from 2000–2006, and studies concur that the main causative genotype was GII.4 [17], [23]. The finding that the only genotype detected in this study in nursing homes and in cases of nosocomial infections was GII.4 was unsurprising, given that in the last decade this genotype has been the main cause of outbreaks in institutions (hospitals, nursing homes, prisons…) worldwide [8], [23]–[26]. We found GII.4 (capsid) associated with GII.P4 (polymerase) in most cases detected in 2009–2011. However, in 2012 this combination was rapidly replaced by GII.Pe/GII.4, due to the emergence of the Sydney-2012 variant [27].

Strains with GII.3 capsid were almost only detected in children, as it has been described in other countries [28]–[30]. NoV GII.3 circulation was intense in the last decades of the last century [9], [30], which, together with high cross-reactivity and limited evolution of GII.3 epitopes, could explain why the incidence of NoV infection is currently scarce in the adult population [28], [31]. The GII.3 strains detected in Gipuzkoa were found in combination with discordant polymerase genotypes, as previously reported [13], [28], [29].

Intergenotype recombination is one of the main mechanisms of NoV GII.3 evolution, and there have been reports of the association of GII.3 (capsid) with polymerases of several genotypes, two of which—GII.P21 (previously GII.b) and GII.P12—were widely described after 2000 [30], [32]. Interestingly, in the present study, genotype GII.3 strains (capsid) associated with GII.P16 (GII.P16/GII.3), were detected after September 2012, partially displacing the classical recombinant GII.P21/GII.3 strain. To our knowledge, this new recombinant GII.P16/GII.3 strain has been previously reported in only two infants admitted to Dhaka Hospital (Bangladesh) in 2011 and 2012 [33]. Its detection in the Basque Country (Spain) suggests global dissemination within a short period of time. Other capsid genotypes (GII.2, GII.6, GII.12 and GII.13) were detected sporadically (6.1%). These are globally distributed genotypes [9], [25], [26], [34], whose prevalence has generally been low in the last few years, with some exceptions in specific geographical areas [9], [24], [26].

Inter- and intragenotypic recombination is one of the main mechanisms of NoV evolution [11], [32], [35]. In the present study, the proportion of intergenotypic recombinant strains was high (31.5%). A recent review that analyzed 803 NoV strains corresponding to 11 studies reported that 26.5% of the detected strains were intergenotypic recombinants [9]. In 2010, we detected a rare recombinant strain, GII.P16/GII.13; to our knowledge, this combination had not previously been reported in the literature but some sequences were included in GenBank from strains isolated in Germany and India (i.e. KC832470.1 and AB757783.1), with which our strain shared a nucleotide identity >98.5% in the analyzed ORF1/ORF2 junction region (1028 bp and 939 bp, respectively).

Since the end of the last century, several GII.4 variants have emerged, some of which have spread worldwide or over large geographical regions, increasing the number of sporadic episodes and outbreaks of AGE [9]–[11]. In the Basque Country, the temporal distribution of variants closely followed that described in other parts of the world. The Den Haag 2006b variant, which was globally dominant in 2007-mid-2009, predominated in Gipuzkoa in 2009. The substantial increase in NoV activity in fall-winter 2009–2010 in this region was due to the emergence of the New Orleans-2009 variant, which also caused an increase in AGE cases in other regions, such as Scotland [25]. In Gipuzkoa, the New Orleans-2009 variant predominated in 2010 and 2011, but was replaced by the new Sydney-2012 variant in the second half of 2012. Sydney 2012 was detected in our region from February 2012 but its dissemination was not associated with epidemic activity in the community until seven months later. A similar tendency was observed in Denmark, where this variant was detected in sporadic cases and some outbreaks from January 2012, but did not become dominant until 10 months later [36]. A few months after the emergence of the Sydney-2012 variant, and before its epidemic spread, we sporadically detected recombinant New Orleans-2009/Sydney-2012 strains, which have recently been reported in Italy [37].

In conclusion, the present study documents the genetic diversity of the genogroup II NoV circulating in the Basque Country (Spain) in 2009–2012. This circulation showed similarities to that in other parts of the world but also some regional particularities. Among the nine different genotypes detected, intergenotypic recombinants, some of which have been reported in other countries only recently, were an important part of them, highlighting the role of recombination in the evolution of NoVs. The detection of genotypes and emerging variants shortly after the first reports (and previously only detected in geographically remote countries) demonstrates the ability of these viruses to spread rapidly. Close surveillance of the distribution and dynamic of the circulation of strains with the potential to spread is therefore required.
